# Evaluation of Insecticidal Effects of Plants Essential Oils Extracted from Basil, Black Seeds and Lavender against *Sitophilus oryzae*

**DOI:** 10.3390/plants10050829

**Published:** 2021-04-21

**Authors:** Nadi Awad Al-Harbi, Nagy M. Al Attar, Dalia M. Hikal, Salwa E. Mohamed, Arafat Abdel Hamed Abdel Latef, Amira A. Ibrahim, Mohamed A. Abdein

**Affiliations:** 1 Biology Department, University College of Tayma, University of Tabuk, Tabuk 71411, Saudi Arabia; nalharbi@ut.edu.sa; 2 Zoology Department, Faculty of Science, Al Azhar University, Cairo 11884, Egypt; nagyalatar@yahoo.com; 3 Nutrition and Food Science, Home Economics Department, Faculty of Specific Education, Mansoura University, Mansoura 35516, Egypt; dr.daliahikal@mans.edu.eg; 4 Department of Molecular Biology, Genetic Engineering and Biotechnology Research Institute (GEBRI), University of Sadat City, Sadat City 32897, Egypt; salwa.khedr@gebri.usc.edu.eg; 5 Biology Department, Turabah University College, Turabah Branch, Taif University, P.O. Box 11099, Taif 21944, Saudi Arabia; 6 Plant Protection and Biomolecular Diagnosis Department, Arid Lands Cultivation Research Institute, City of Scientific Research and Technological Applications, New Borg El-Arab, Alexandria 21934, Egypt; amiranasreldeen@yahoo.com; 7 Biology Department, Faculty of Arts and Science, Northern Border University, Rafha 91911, Saudi Arabia

**Keywords:** *Sitophilus oryzae*, cytochrome gene, detoxification, antioxidant activity, essential oils, phytochemical constituents

## Abstract

The risk of using synthetic insecticides to the environment, human health, and the emergence of new genera of pests resistant to that kind of drugs, have led to attention in natural compounds. The present study aimed at evaluating the insecticidal activity of 0.25–6 mg/cm^2^ of basil (*Ocimum basilicum*), black seeds (*Nigella sativa*), and lavender (*Lavandula angustifolia*) essential oils (EOs) against one of the major stored product pests, *Sitophilus oryzae* (L.). This was done by assessing mortality and repellent percentage assay in the adult stage, as well as analysing up and down-regulated genes associated with toxicity effect of selected EOs. The three studied EOs showed a toxic effect on *S. oryzae*; where *O. basilicum* and *L. angustifolia* EOs explicated 100% mortality at 6 mg/cm^2^ after 48 and 24 h, respectively. The highest repellence activity was recorded for O. *basilicum* EO at 0.75 mg/cm^2^ with value 82.3% after exposure time 5 h. In the highest dose (6 mg/cm^2^), the maximum up-regulated expression level of detoxification DEGs genes (CL1294 and CL 8) and cytochrome p45o gene (CYP4Q4) in *Lavandula angustifolia* EOs exhibited 8.32, 6.08, and 3.75 fold changes, respectively, as compared with 4.76 fold at 10 ppm malathion and 1.02 fold change in acetone control.

## 1. Introduction

In 2019, the latest forecast cereal production recorded an increase of 1.2% from 2018, to about 2.685 million tons, according to FAO [1]. Rice is one of the most important crops for almost all of the world especially developing countries [2]. Rice is considered a principal diet for more than two billion people because it has minerals, vitamins, fibers, and carbohydrates [3]. According to storage conditions, rice grains could be attacked by different insects and pests such as *Sitophillus oryzoe* L., which can be affected in quantity and nutritional quality [4].

In developing countries, loss of cereal foods is considered the major problem due to pest infections through storage [5,6,7]. Many insects, mites, and fungi attack stored cereals and led to a decrease its quality in addition to losses from 9 to 20% [8]. To overcome product losses by pests, synthetic pesticides were been used since 1960 [9]. Using these synthetic pesticides is a critical crisis especially in increasing insect resistance and the harmful impact on humans and the environment [10,11,12]. Additionally, insecticides have a dangerous impact on the malformation of food grains because of residues and harmfulness of synthetic chemicals to non-target organisms in surroundings. Besides, the use of these insecticides resulted in chromosomal aberration and genetic mutation in both plant and human [13,14,15]. As a result of this damage to the environment, researchers have recently thought about considered using safe alternatives for example plant extracts and plant essential oils [16].

Plant essential oils recently have been used as a biological control for insects and pests through their usages as anti-fungal, anti-microbial, and in allelopathic potentialities (herbicide uses) [11,17]. The importance of essential oils as insecticides and pesticides due to their valuable characteristics [18,19,20,21] less persistence of essential oils in air and environment due to their high volatility and degradation sensitivity to temperature and UV and sunlight [22]. Regarding their important characteristics, essential oils considered safer and more eco-friendly than synthetic pesticides and insecticides; additionally, essential oils are with a lower toxicity for mammalians [23]. Volatile plant essential oils have a mixture of 20–60 constituents which gave their characteristic odor and flavor [24]. A lot of essential oils have a toxicity effect against different types of stored food insects and pests [25]. Plant essential oils contain monoterpenoid compounds that toxic for insects through destroying the nervous system [26].

Basil (*Ocimum basilicum*) essential oil was used as a disinfectant against *Ephestia kuehniella*, *Tribolium castaneum,* and rice weevil (*Sitophilus oryzae*) [27], also basil oil had insecticidal properties and antifungal activity [28]. Black seeds (*Nigella sativa*) essential oil had a fumigant effect and repellence activity against *Tribolium castaneum* larva and adults, even with low concentrations [29]. Lavender (*Lavandula angustifolia*) essential oil chemical constituents had a wide range of toxicity against fungi, bacteria, insects, and pests [30]. [31] showed the repellence activity of *L. angustifolia* essential oil to *Sitophilus oryzae* L., *Rhizopertha dominica* F., and *Tribolium castaneum* Herbst. Different chemical compounds in *Lavandula* essential oil gave different efficiency and bioactivity control for pests and insects [32].

In recent years, there are a variety of detoxification enzymes encoded in insect genomes, including glutathione S-transferase (GST), cytochrome oxidase P450 (CYP), and carboxylesterase (CarE, also known as CCE/EST/CES) [33,34] that have potential activities in xenobiotic compounds detoxification. Furthermore, transcriptional regulation of gene expression of GST, CarE, and AchE enzymes in insects after insecticide application has been utilized for understanding the insect response and insecticidal mechanism to various xenobiotic compounds stresses [35,36]. However, molecular mechanisms remain unclear for the insecticidal activities of the essential oils from *Ocimum basilicum*, *Nigella sativa,* and *Lavandula angustifolia* medicinal plants against the stored product insect pest *Sitophilus oryzae* (L.). According to the economic importance of rice and its loss in quantity and quality by *S. oryzae*, the objective aim of this study was: (1) to assess the fumigant and insecticidal activity of three botanical essential oils from basil, black seeds, and lavender against *Sitophilus oryzae* adult stage; (2) screening chemical composition of the studied essential oils using GC-MS; and, finally (3) estimation significant roles of the three crucial detoxification enzymes related genes (Cl8, CYP 4Q4, and CL1294) in the response of *Sitophilus oryzae* insect to *Ocimum basilicum*, *Nigella sativa,* and *Lavandula angustifolia* essential oils.

## 2. Materials and Methods

### 2.1. Insect, Collection, Rearing and Treatment 

Briefly, the insect strain utilized in this study (*Sitophilus oryzae*) was collected originally from a rice store bin in Beheira Governorate, Egypt. It has been reared at 28 °C on 95% corn seeds and 5% brewer′s yeast, 65% RH. Insects were sub-cultured from the laboratory colony, and specific life stages were removed. Mixed-sex adults were collected at 3–7 days post eclosion. 

### 2.2. Isolation of Essential Oils

In this study, three herbal and medicinal plant species; basil flowers (*Ocimum basilicum*), seeds of black seeds (*Nigella sativa*), and lavender flowers (*Lavandula angustifolia*), were selected according to their ethnomedicinal importance and literature survey and were collected from Botanical Gardens and Ornamental Plants Department, Horticulture Research Institute (HRI) Institute, Agricultural Research Center, Egypt, and the botanical identification was conducted by Prof. Ibrahim Mashaly, Professor of Flora and plant Ecology at Department of Botany, Faculty of Science, Mansoura University, Egypt in July 2019. Plants were shadow dried at room temperature and 50 g were packed and stored at −4 °C. Oils were extracted through steam-distillation utilizing a Clevenger-type apparatus [37]. After a distillation time of 8 h, 50 g of each plant of the dried material yielded nearly 2 mL oil. The distillation was repeated to obtain the required oil quantity for research purposes.

### 2.3. Preliminary Phytochemical Analysis of EOs

Phytochemical characteristics of the essential oils isolated from the selected basil flowers, black seeds, and *lavender* leaves were illustrated using the following tests:

#### 2.3.1. Screening for Carbohydrate Test

First, 1 mL of EOs of selected plants was added to 1 mL of Benedict’s reagent, and the mixture was then heated for 2 min in a boiling water bath. A green solution indicated the presence of reducing sugar.

#### 2.3.2. Screening for Glycosides (Keller Kilianin Test)

Then, 5 mL of isolated essential oils were added with 2 mL of glacial acetic acid, few drops of ferric chloride solution and 1 mL of concentrated sulfuric acid, leading to brown ring formation at the interface, indicating the presence of glycosides.

#### 2.3.3. Screening for Terpenoids (Salkowski Test)

Then, 5 mL of isolated essential oils were taken from the plants, and 2 mL of chloroform and 3 mL of concentrated sulfuric acid were then added. If a reddish-brown layer formed at the junction of the two solutions, the presence of terpenoids was indicated.

#### 2.3.4. Screening for Steroids

Then, 1 mL of extracted essential oils was dissolved in 10 mL chloroform, followed by the addition of an equal volume of sulfuric acid. Red color appeared in the upper and yellow color with green fluorescence developed in the sulfuric acid layer, revealing the presence of steroids.

#### 2.3.5. Screening for Tannins

Then, 2 mL of essential oils extracted from three selected plants were added to a few drops of 1% lead acetate. A yellowish precipitate was developed as a result of tannins presence in the solution.

### 2.4. Bioassay

#### 2.4.1. Insect Mortality 

To determine the mortality effect of the studied EOs against *Sitophilus oryzae* against *Sitophilus oryzae*, contact effect was evaluated on filter paper discs through treatment of a Whatman No.1, 8 cm diameter, area = 54.4 cm^2^). The filter paper discs were treated with 0.5 mL of acetonic solutions of EOs, and in the control treatment, the filter paper disc was treated with an equal volume of acetone. The four replicates for each treatment from different EOs solutions were tested corresponding to their doses (2, 4, and 6 mg/cm^2^) compared with positive control treatment and 10 ppm of the chemical standard malathion. Treated and control filter paper halves were air-dried for 1 h for solvent evaporation. Then, 10 unsexed adult beetles (3–7 days old) were released in the filter paper center, and the lid was then sealed with Parafilm. The experiment was run in the dark at 28 ± 2 °C and 65 ± 5% RH. The dead insects were counted at 3, 6, 12, 24, and 48 h post-application. Insects without response to the gentle touch of a small probe were considered dead [38] and data were corrected using Abbott’s formula [39].

#### 2.4.2. Insect Repellence (Filter Paper Disc Bioassay)

A bioassay system was used to evaluate the activity of the three studied EOs using the area preference method [40]. A filter paper disk (Whatman No. 1, 8 cm diameter, area = 54.4 cm^2^) was divided into 2 halves, one of which was then treated with 0.5 mL of acetonic solutions of EOs, and the control half was treated with an equal volume of acetone. The four replicates from EOs acetone solutions were tested corresponding to the doses of 0.25, 0.5, and 0.75 mg/cm^2^ compared with the positive control treatment (malathion, 4 ppm) as chemical control. The treated and control filter paper half were air-dried for about 60 min, allowing solvent evaporation. After that, the paper disc was joined and fixed on the bottom of a Petri dish, and 10 3–7 days old, unsexed adults were then released in the center of both halves. The experiment was maintained at 28 ± 2 °C and 65 ± 5% RH in a dark place. Repellency percentage was recorded at 1, 2, 3, 4, and 5 h depending on the number of insects noticed on both treated and untreated halves. Percentage repellency was calculated by the following equation:

(1) $$\mathrm{Repellence}\;(\%)=\;\left(Nc-Nt\right)/\left(Nc+Nt\right)\;\times \;100,$$

where *Nc* = the number of insects on the untreated half, and *Nt* = the number of insects on the treated half, after the time exposure.

### 2.5. Chemical Composition of EOs by GC-MS

For determination of the chemical composition of basil, black seeds, and lavender EOs, gas chromatography-mass spectrometry (GC-MS) analysis was conducted using GC-2010 Shimadzu capillary gas chromatography directly coupled to the mass spectrometer system (GC-MS–model QP 2010; (Shimadzu, Kyoto, Japan) DB-c18 column under following conditions: Injector temperature is 250 °C. Oven temperature program: initial temperature 30 °C for 2.0 min, ramp 2.0 °C/min to 250 °C, hold for 5.0 min. MS source temperature is 200 °C, electron energy is 70 eV; the carrier gas was helium at a flow rate 1.4 mL/min; 1 µL of each diluted sample in n-hexane (1:1, *v/v*) was injected. EI spectra were scanned from 43.00 to 600 *m/z* Identification of peaks through NIST mass data search libraries and the highest REV and for similarity indicators hits. The components of the sample were identified based on a comparison of their relative indices and mass spectra by computer matching with WILEY and National Institute of Standards and Technology (NIST08) libraries provided with the computer controlling GC-MS system [41].

### 2.6. Phytochemical Analysis of Three Selected EOs Antioxidant and Free Radical Scavenging Capacity 

#### 2.6.1. Estimation of Total Phenolic Content (TPC)

Total phenolic content in three studied essential oils was determined using the Folin Ciocalteu (FC) method of Su [38] method with slight modifications as prepared at a concentration of 1 mg/mL. A calibration curve was prepared using gallic acid (1–0.05 mg/mL). Then, 1 mL of *O. basilicum, N. sativa,* and *L. angustifolia* EOs or gallic acid was added to 0.5 mL of FC reagent and was vortexed well, followed by standing at room temperature for 5 min. Then, 1 mL, 7.5% *w/v,* of sodium carbonate, was added and maintained for 1 h at room temperature. The absorbance was measured at a wavelength of 760 nm. TPC was expressed as mg gallic acid equivalents (GAE)/g sample.

#### 2.6.2. Estimation of Total Flavonoid Content (TFC)

Total flavonoid content in three studied EOs was determined by the colorimetric method [42]. About 300 μL of three studied EOs were mixed with 30 μL of 16% NaNO_2_. The mixture was then maintained at room temperature for 1 h, and 200 μL of NaOH solution (1 M) was added, followed by 60 μL of 10% AlCl_3_, and 700 μL H_2_O was then added to the mixture. The volume of the mixture was completed up to 1 mL with distilled water. The absorbance of the mixture was measured at 510 nm. The results were expressed as mg of rutin equivalent per gram of fresh weight (mg RE/g FW).

#### 2.6.3. Antioxidant Activity by Free Radical Scavenging Capacity (DPPH)

To assay the free radical scavenging capacity of the investigated EOs on the stable free radical 1, 1-Diphenyl-2-picrylhydrazyl (DPPH) was determined according to [42]. Typically, 100 μL of DPPH (0.004% prepared in methanol) were mixed with 0.1 mL of tested EOs or vitamin C (reference). The plate was mixed by shaking, wrapped with aluminum foil, and then maintained for 30–60 min at 25 °C in a dark place. colors ranged from deep violet to light yellow, and the decrease in absorbance was measured at 517 nm. The DPPH was calculated from the following equation:

(2) $$\mathrm{DPPH}\;\mathrm{scavenged}\;(\%)=\frac{\left(AC-AE\right)}{AC}\;\times \;100,$$

where *AC*: The mean of absorbance of the negative control; *AE*: The mean of absorbance of extract sample or standard.

### 2.7. Molecular Response of Insect to Botanical Pesticides

The molecular mechanism of botanical EOs pesticides and their mode of action was evaluated by studying the transcriptional expression levels for proteasome subunit alpha type-5 (CL8), arylalkylamine N-acetyltransferase (CL1294), and cytochrome (CYP 4Q4) gene in *S. oryzae* adult to the applied essential oils using the quantitative real-time polymerase chain reaction (qRT-PCR) analysis.

#### 2.7.1. Total RNA Isolation from *Sitophilus oryzae*

The three replicates from *S. oryzae* adult samples (2–7 days) (without treatment as control and other treated with three selected EOs) after 1 and 2 h exposure time of treatment were used for total RNA extraction using about 0.1 g from each replicate through using Tripure total RNA extraction reagent, according to the manufacturer’s instructions. RNA concentration and purity were recorded by using NanoDrop™ 2000 (A260/A280).

#### 2.7.2. cDNA Synthesis and Real-Time PCR

Reverse transcription reactions (RT-PCR) for the total RNA were performed. The volume of reaction was 20 μL contained 2.5 μL from oligo primer (10 pmL/μL), 2.5 μL

RNA (2 μg), 2.5 μL 5X buffer, 2.5 μL MgCl_2_, 2.5 μL dNTPs (2.5 mM), and 0.2 μL reverse transcriptase (MLV, Fermentas, USA) (5 Unit/μL). RT-PCR amplification was performed in a thermal cycler (Promega, Germany), programmed at 42 °C for 1 h and the enzyme was killed at 72 °C for 10 min. The cDNA was then stored for further studies at −20 °C [43].

#### 2.7.3. Quantitative Real-Time –PCR (qRT-PCR)

qRT-PCR analysis was performed for three insecticidal detoxification genes (CL8, CL1294, and CYP4Q4) in *S. oryzae* adults treated with three selected EOs using SYBR Green Master Mix method utilizing a Rotor-Gene 6000 real-time PCR detection system (Qiagen, Germany). The primer sequences used in qRT- PCR mentioned in Table 1 were designed corresponding to the CL8, CL1294, and CYP4Q4 genes. β-Actin gene was used as an internal control gene. For each gene three replicates were used for qRT-PCR reaction; reaction volume of 25 μL contained 12.5 μL of 1×SYBR GREEN PCR mix (TaKaRa Code: RR041A), 1 μL 1:10 diluted cDNA templates, 1 μL sets of each primer and 9.5 μL Rnase free double distilled water according to the manufacturer’s protocol. RT-qPCR was amplified in 3-step and 45 cycles at 95 °C for 15 s, and 60 °C for 30 s and 70 °C for 30 s. Data of qRT-PCR were collected as CT (PCR cycle number where fluorescence is detected above the threshold). The CT of each sample was used to calculate ΔCT values (target gene CT subtracted from 18sRNA gene CT). The relative gene expression of studied genes was determined using the 2^−ΔΔCt^ method [44,45]. Data from PCR runs were analyzed in the MyiQ optical system software version 1.0 (Bio Rad Laboratories Inc., Hercules, CA, USA). The expression quantity of expressed genes was calculated according to the Sigma Plot version 9.0 software (Systat Software Inc.: San Jose, CA, USA).

### 2.8. Statistical Analysis

The results are presented as means ± standard deviation (±SD). Data were analyzed using the statistical package for social sciences (SPSS) 16 and were evaluated by analysis of variance (ANOVA). Duncan’s test was used for comparisons among different treatments. Statistical differences were considered significant at the *p* < 0.05 level 3.

## 3. Results

### 3.1. Preliminary Phytochemical Screening Analysis of EOs

According to preliminary phytochemical screening results of *O. basilicum*, *N. sativa* and *L. angustifolia* EOs, Table 2 shows the presence of phytochemical constituents in the selected botanical EOs with antioxidant activities such as glycosides in *O. basilicum*, *N. sativa* extracts, steroids, terpenoids, tannins and sterols compounds in *O. basilicum*, *N. sativa* and *L. angustifolia* EOs.

### 3.2. GC-MS Analysis of Essential Oils

In this study, qualitative analyses of basil, black seeds, and lavender EOs by using GC-MS showed the presence of many bioactive gradients, which may have a role in insecticidal effects. The peak area (%) and the retention time of the chemical compounds in the analyzed oils were enumerated in its chromatogram.

#### 3.2.1. Analysis of *O. basilicum* (basil) EOs

**Scheme 1:**
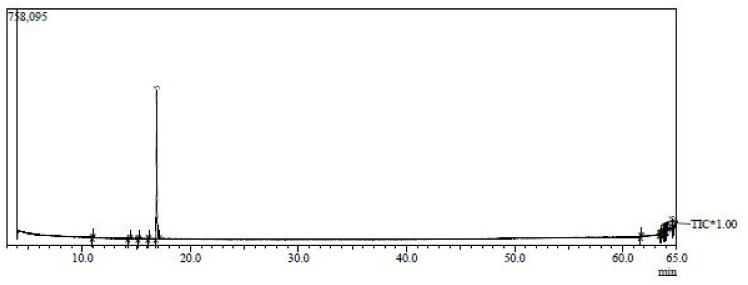
GC-MS chromatogram of O. *basilicum* EOs.

Chemical analysis performed by GC-MS analysis of *O. basilicum* EOs was demonstrated in Scheme 1 that is characterized by the presence of many biologically important compounds listed in Table 3 such as linalool Eugenol, (1S)-camphor, Octamethylcyclotetrasiloxane and linalyl acetate compounds that are referenced with insecticide properties constituents.

#### 3.2.2. Analysis of *N. sativa* (Black Seeds) EOs

**Scheme 2:**
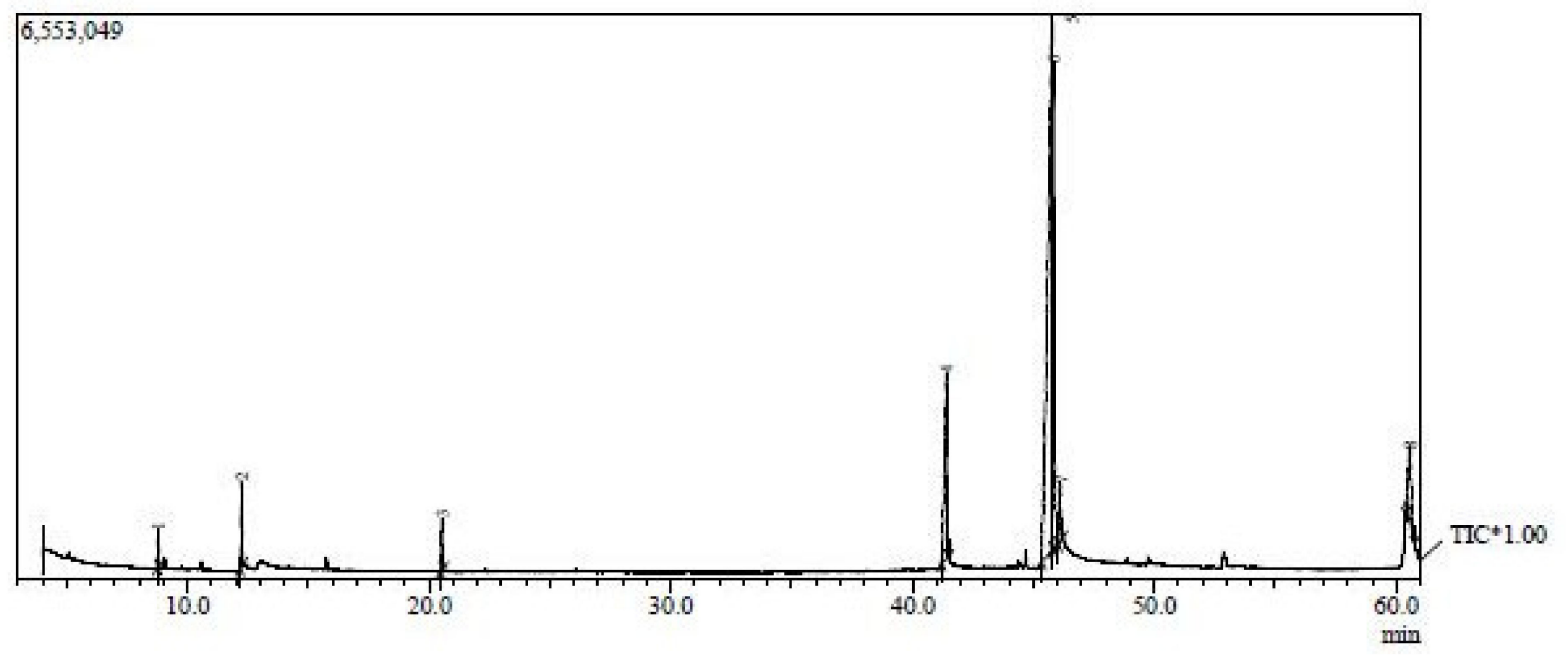
**G**C-MS chromatogram of *Nigella sativa* Eos.

Chemical analysis performed by GC-MS analysis of *N. sativa* EOs was illustrated in Scheme 2, which is characterized by the presence of many biologically important compounds listed in Table 4 such as α-Thujene, p-cymene, Palmitic acid, Linoleic acid, Erucic acid, and Trielaidin compounds that referenced with insecticide properties constituents.

#### 3.2.3. Analysis of *L. angustifolia* (Lavender) EOs

**Scheme 3:**
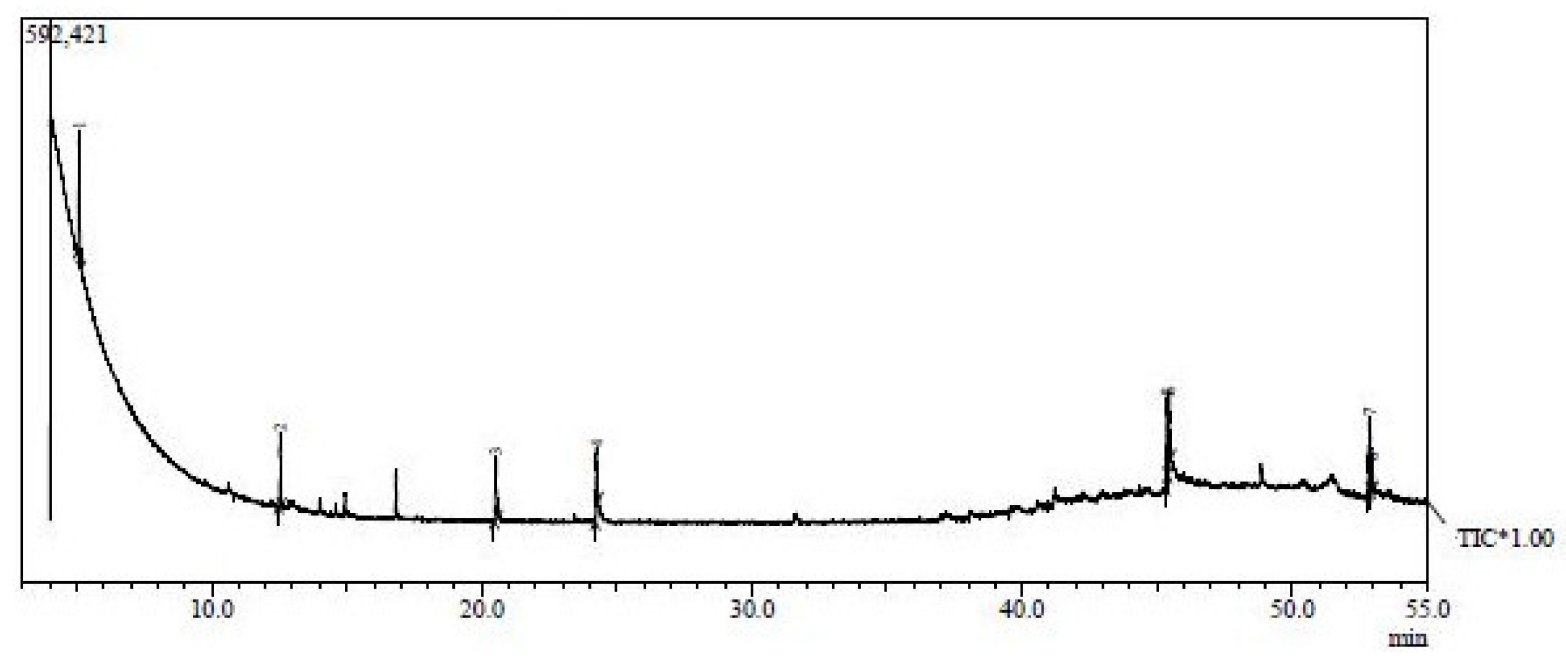
GC-MS chromatogram of *L. angustifolia* EOs.

Chemical analysis performed by GC-MS analysis of *L. angustifolia* EOs was represented in Scheme 3, which is characterized by the presence of many biologically important compounds listed in Table 5 such as hexanal, Eucalyptol, Lavandulyl acetate, Eugenol, Bicyclo[10.1.0]tridec-1-ene, Linoleic acid, and cis-11-Eicosenoic acid compounds that referenced with insecticide properties constituents.

### 3.3. Antioxidant Activity and Free Radical Scavenging Capacity

Among the non-enzymatic antioxidants parameters, the levels of total phenolic content, total flavonoid content, and free radical scavenging capacity (DPPH) were measured in the study and the data in Table 6 show significant contents of TPC and TFC and DPPH capacity in three studied EOs. The highest content from total phenol and total flavonoid was 31.4 mg GAE/g and 17.6 mg QE/g in basil EOs. Additionally, a maximum DPPH capacity 18.9% was reported in basil essential oil.

### 3.4. Mortality Bioassay against Adult

Overall, mortality percentages in *S. oryzae* adult stage increased with increasing concentrations (2, 4, and 6 mg/cm^2^) and exposure time (3, 6, 12, 24, and 48 h) after using essential oils of basil, black seeds and lavender compared to control (10 ppm malathion).

**Figure 1:**
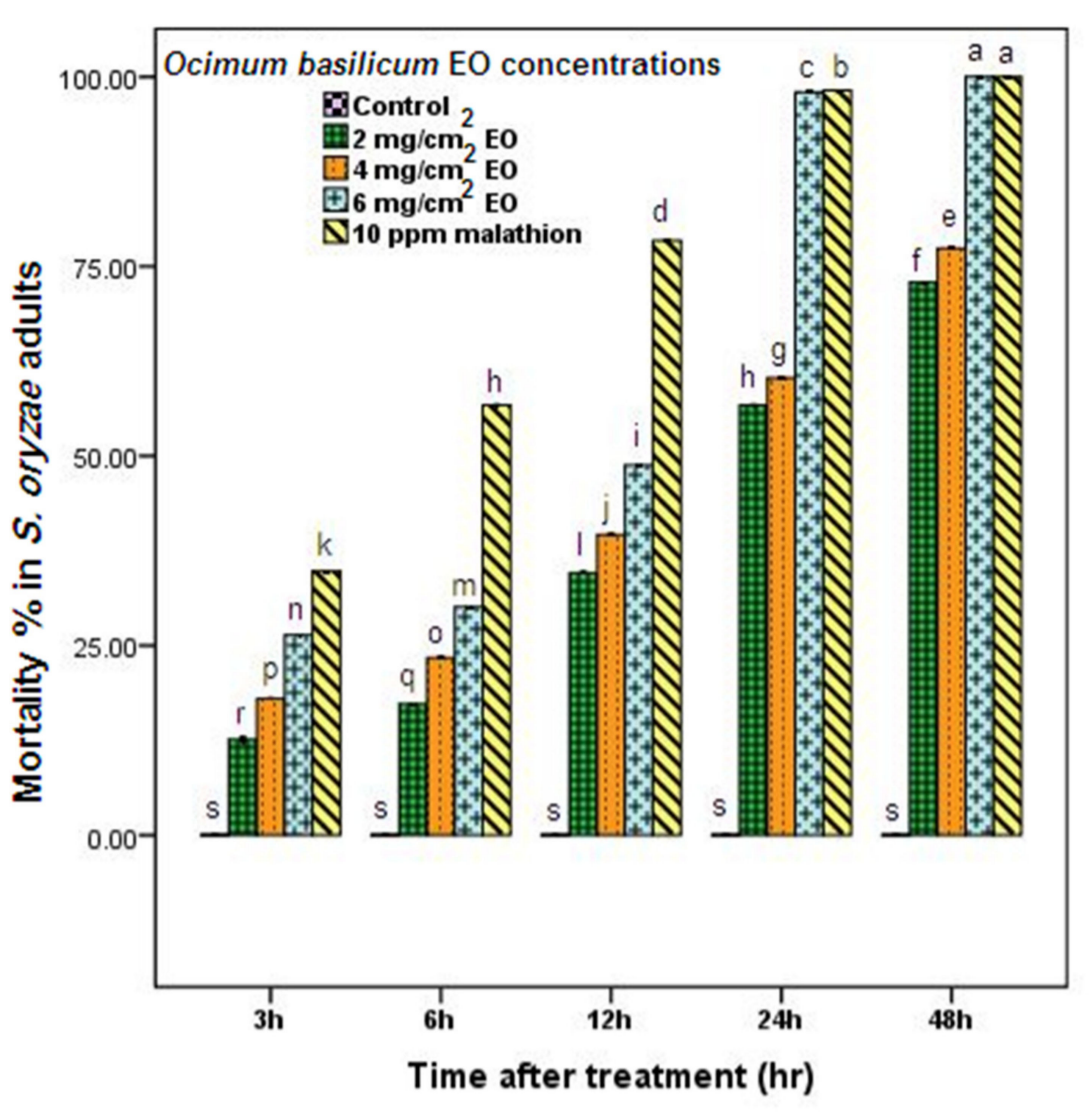
Impact of different concentrations (2, 4 and 6 mg/cm^2^) from *O. basilicum* EOs on mortality of *S. oryzae* adults at 3, 6, 12, 24, and 48 h exposure times (mean ± SD) compared with standard chemical pesticide (10 ppm malathion). Different letters indicate significant differences between different treatments at *p* < 0.05; LSD = 0.036.

For *O. basilicum* EOs, the obtained results represented in Figure 1 illustrated that the highest significant value of mortality 100% using concentration 6 mg/cm^2^ at 48 h exposure time, compared to control (10 ppm malathion), followed by 92.2% at 6 mg/cm^2^ after 24 h exposure time. On the other hand, there was no mortality in any of the untreated control.

**Figure 2:**
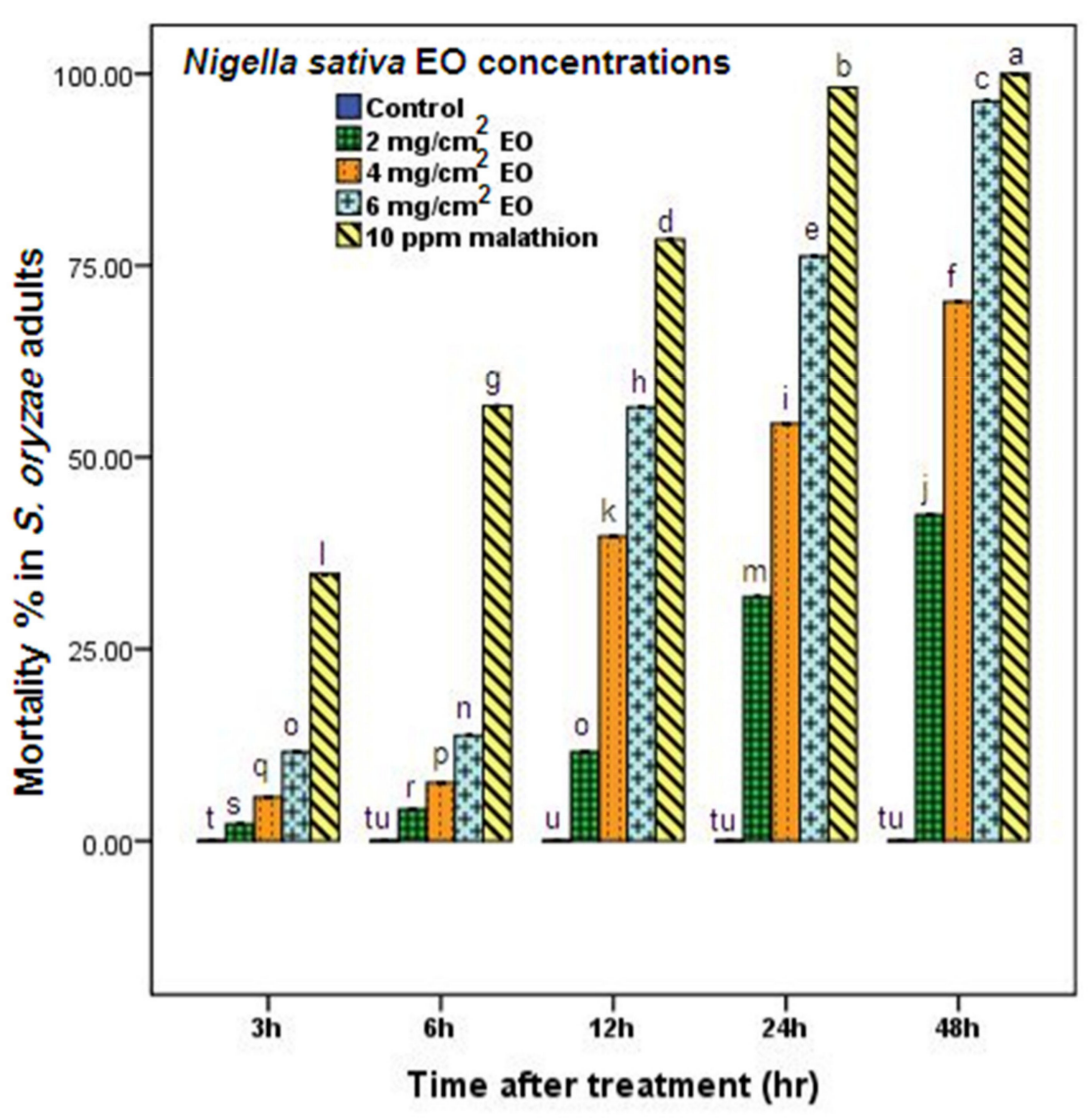
Impact of different concentrations (2, 4, and 6 mg/cm^2^) from *N. sativa* EOs on mortality of *S. oryzae* adults at 3, 6, 12, 24, and 48 h exposure times (mean ± SD) compared with standard chemical pesticide (10 ppm malathion). Different letters indicate significant differences between different treatments at *p* < 0.05; LSD = 0.016.

For *N. sativa* EOs, the data presented in Figure 2 showed that the highest significant mortality of *S. oryzae* adult stages was recorded at concentration 6 mg/cm^2^ at all exposure times. The highest percentage of mortality of *S. oryzae* was reported at 6 mg/cm^2^ at 48 h with the value of 96.4% followed by the concentration 6 mg/cm^2^ at 24 h with the value of 70.3% compared to control with the value 98.2%.

**Figure 3:**
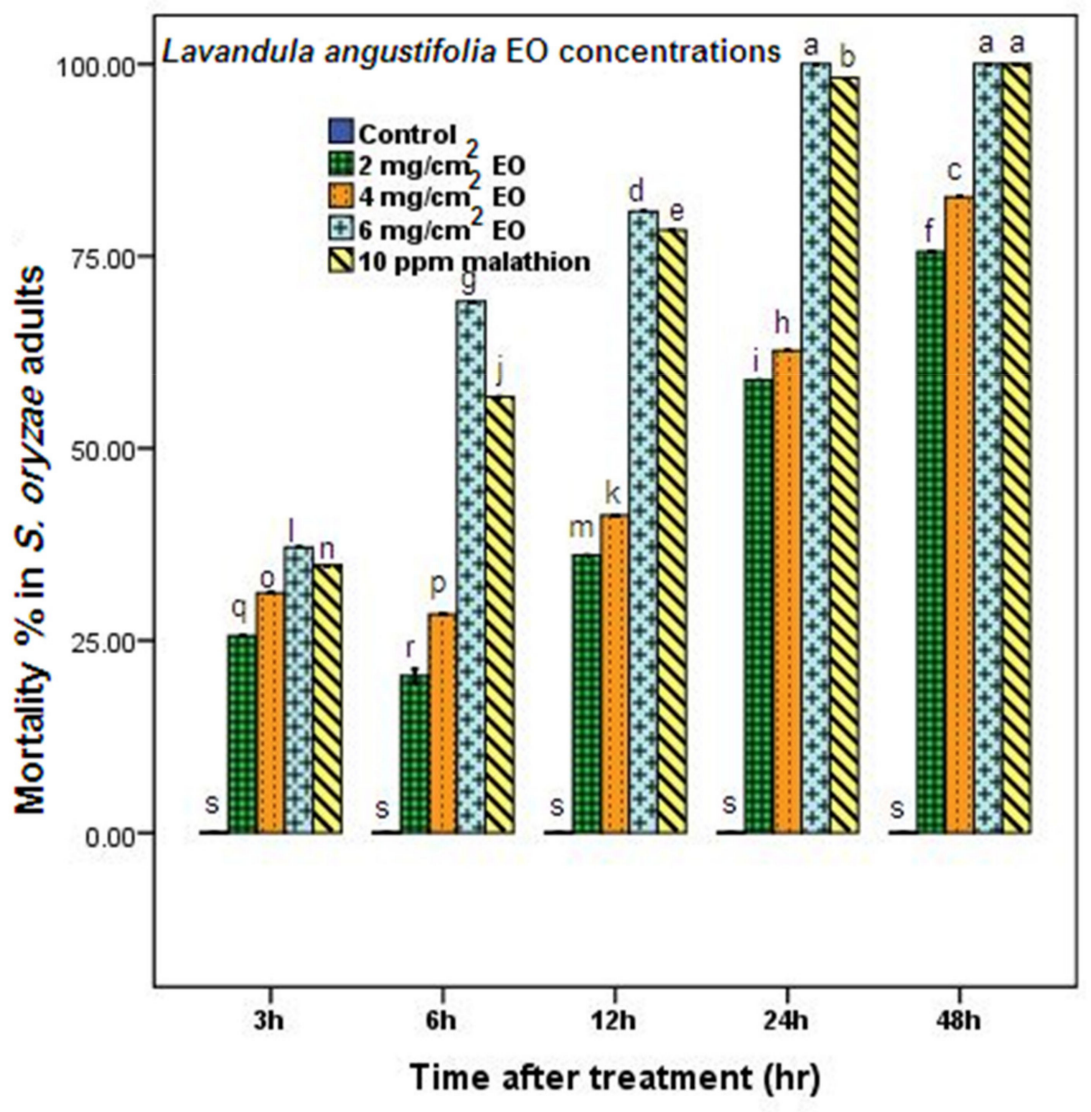
Impact of different concentrations (2, 4, and 6 mg/cm^2^) from *L. angustifolia* EOs on mortality of *S. oryzae* adults at 3, 6, 12, 24, and 48 h exposure times (mean ± SD) compared with standard chemical pesticide (10 ppm malathion). Different letters indicate significant differences between different treatments at *p* < 0.05; LSD = 0.115.

For *L. angustifolia* EOs, the highest significant mortality of *S. oryzae* adult stages was represented in the highest concentration 6 mg/cm^2^ at all exposure times. The highest percentage of *S. oryzae* mortality 100% was recorded at 6 mg/cm^2^ after 48 and 24 h compared to their control values at 48 and 24 h were 100 and 98.2%, respectively, followed by 98.2 and 80.8% for 6 mg/cm^2^ at 24 and 12 h, respectively (Figure 3). Finally, after using three selected EOs against *S. oryzae* mortality; *L. angustifolia* EOs recorded the highest percentage of mortality 100% at exposure time 48 and 24 h, followed by *O. basilicum* recorded the percentage of mortality 100 and 98.2% at 48 and 24 h, respectively.

### 3.5. Repellant Bioassay against Adult

**Figure 4:**
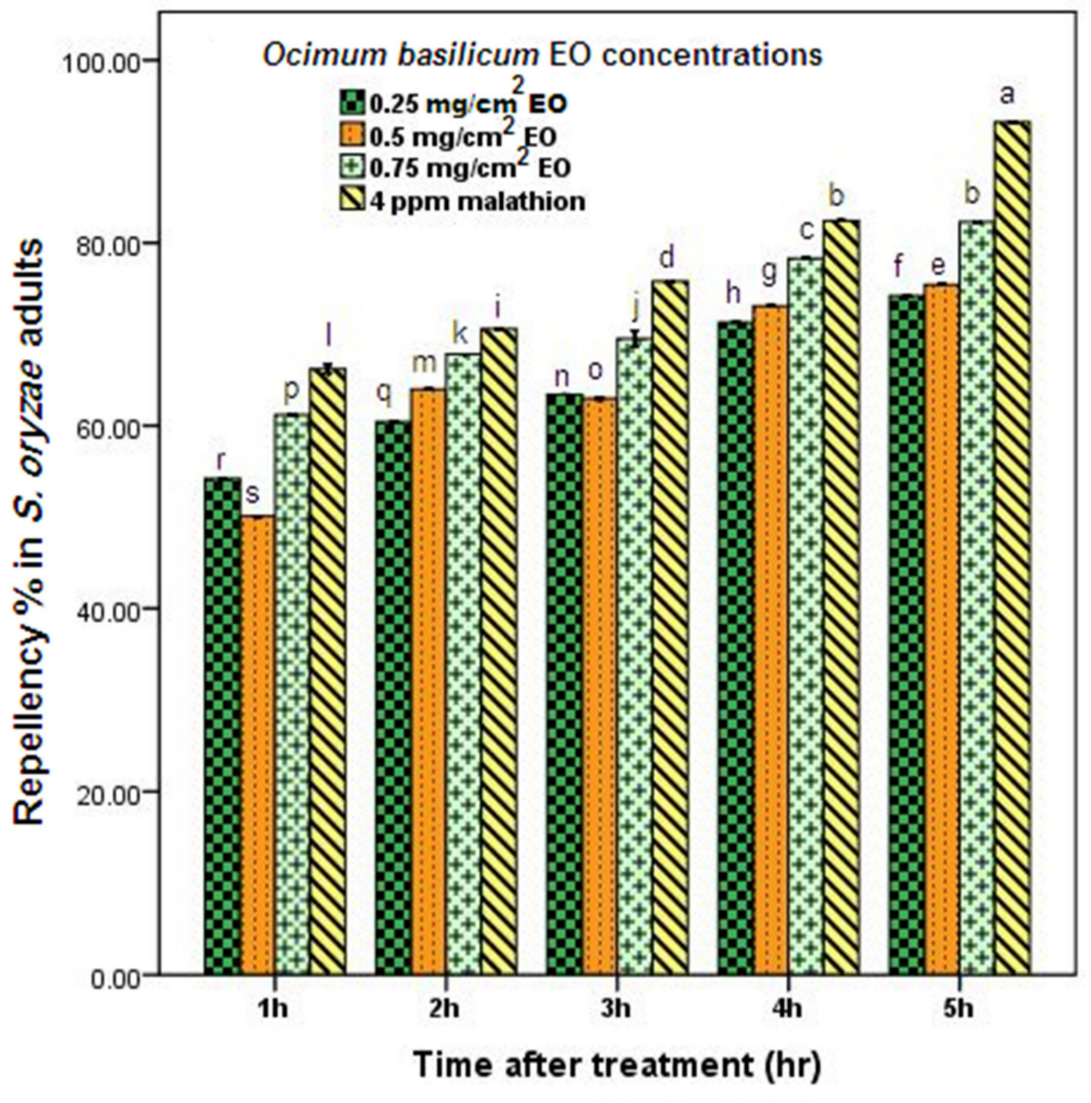
Impact of different concentrations (0.25, 0.5, and 0.75 mg/cm^2^) from *O. basilicum* EOs on repellent % of *S. oryzae* adults at 1, 2, 3, 4, and 5 h exposure times (mean ± SD) compared with standard chemical pesticide (4 ppm malathion). Different letters indicate significant differences between different treatments at *p* < 0.05; LSD = 0.157.

In general, the repellant percentage in *S. oryzae* adult stage increased with increasing the concentrations of three studied EOs and increasing exposure times. Regarding *O. basilicum* EOs results in Figure 4, the highest significant repellency of *S. oryzae* was recorded as 82.3% for 0.75 mg/cm^2^ at 5 h compared to control (4 ppm malathion) at 5 h with the value of 93.2%, followed by 78.3 and 69.7% for 0.75 mg/cm^2^ at 4 and 3 h, respectively, while the lowest significant repellency value was 54.2% at 0.25 mg/cm^2^ *O. basilicum* after 1 h.

**Figure 5:**
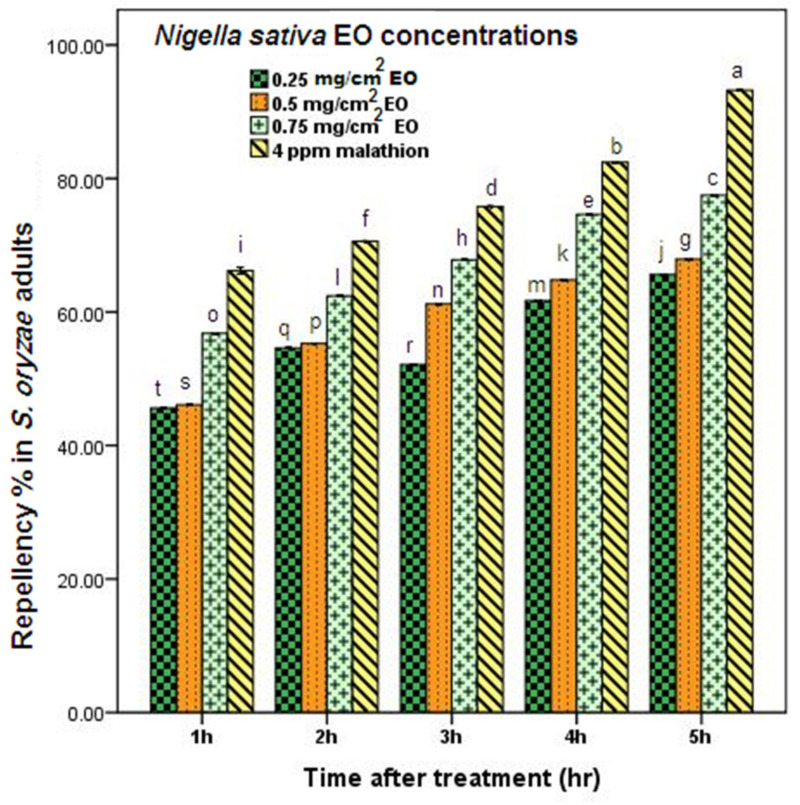
Impact of different concentrations (0.25, 0.5, and 0.75 mg/cm^2^) from *N. sativa* EOs on repellent % of *S. oryzae* adults at 1, 2, 3, 4, and 5 h exposure times (mean ± SD) compared with standard chemical pesticide (4 ppm malathion). Different letters indicate significant differences between different treatments at *p* < 0.05; LSD = 0.127.

For *N. Sativa* EOs, data represented in Figure 5 indicated that the highest significant repellency of *S. oryzae* adult stages was 77.5% at 0.75 mg/cm^2^ after 5 h compared to its control at the same exposure time with the value of 93.2%, followed by 74.6 and 67.7% for 0.75 mg/cm^2^ after 4 and 3 h, respectively, while the lowest significant repellency value was 45.6% at 0.25 mg/cm^2^ EO after 1 h.

**Figure 6:**
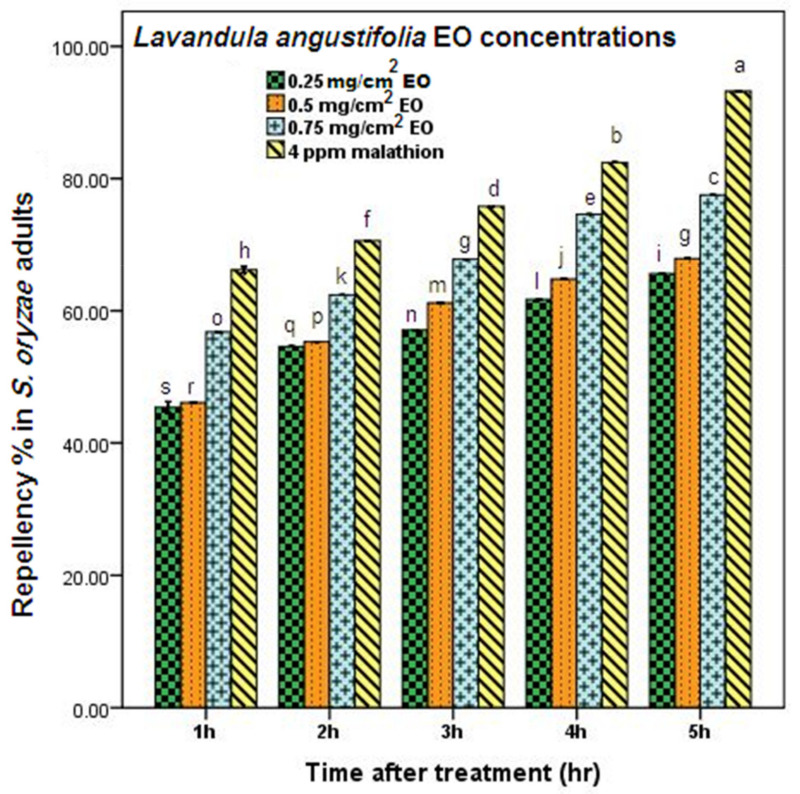
Impact of different concentrations (0.25, 0.5, and 0.75 mg/cm^2^) from *L. angustifolia* EOs and on repellent % of *S. oryzae* adults at 1, 2, 3, 4, and 5 h exposure times (mean ± SD) compared with standard chemical pesticide (4 ppm malathion). Different letters indicate significant differences between different treatments at *p* < 0.05; LSD = 0. 0.157.

For *L. angustifolia* EOs, obtained results in Figure 6 showed that the highest repellency percentage of *S. oryzae* adult stages increasing with increasing the concentration of EOs (0.25, 0.5, and at all exposure times). The highest significant repellence activity of *L. angustifolia* EO was recorded at 0.75 mg/cm^2^ after 5 h with the value of 77.5% after 5 h versus its control (4 ppm malathion) was 93.2% after the same exposure time, followed by 74.6 and 67.8% for 0.75 mg/cm^2^ after 4 and 3 h, respectively. While the lowest repellency value was 45.6% at 0.25 mg/cm^2^. On the other hand, the lowest significant repellency value was 45.6% at 0.25 mg/cm^2^ *L. angustifolia* after 1 h.

These results revealed that *O. basilicum* EOs recorded the highest repellence activity against *S. oryzae* with a value of 82.3% followed by *L. angustifolia* and *N. sativa* EOs.

### 3.6. Gene Expression Analysis

qRT-PCR analysis was performed at two different times after treatment (1 and 2 h) for three detoxification genes of Cytochrome P450 (CYP4Q4) and DEGs genes (CL8 and CL1294) in *S. oryzae* adult stage treated with *O. basilicum, N. sativa,* and *L. angustifolia* EOs as a natural insecticidal agent.

**Figure 7:**
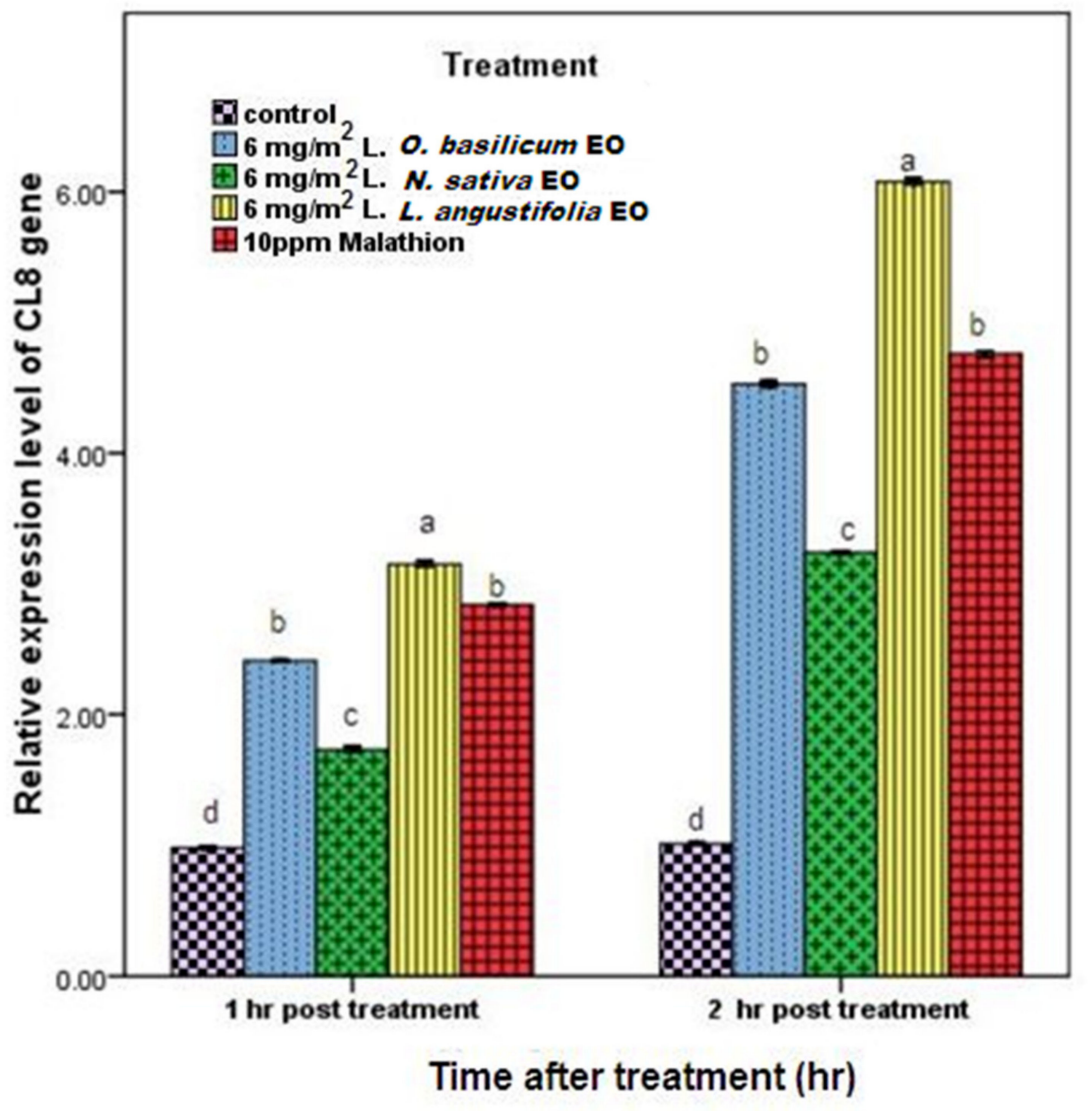
Relative gene expression of DEGs gene (CL8) gene in *S. oryzae* adults after 1 and 2 h treated with 6 mg/cm^2^ of *O. basilicum, N. sativa,* and *L. angustifolia* EOs (mean ± SD) compared with standard chemical insecticide (10 ppm malathion). Different letters indicate significant differences between different treatments at *p* < 0.05.

According to the qRT-PCR results in Figure 7, it showed maximum up-regulated expression level of CL8 gene exhibiting 6.08 fold changes in mRNA in *S. oryzae* adults treated with 6 mg/cm^2^ concentration of *L. angustifolia* EOs for 2 h, followed by 4.76 fold at 10 ppm malathion after 2 h. The minimum expression level reached 1.73 fold in adult insects treated with (6 mg/cm^2^) of *N. sativa* EOs for 1 h as compared with the reference gene (housekeeping gene, β-Actin).

**Figure 8:**
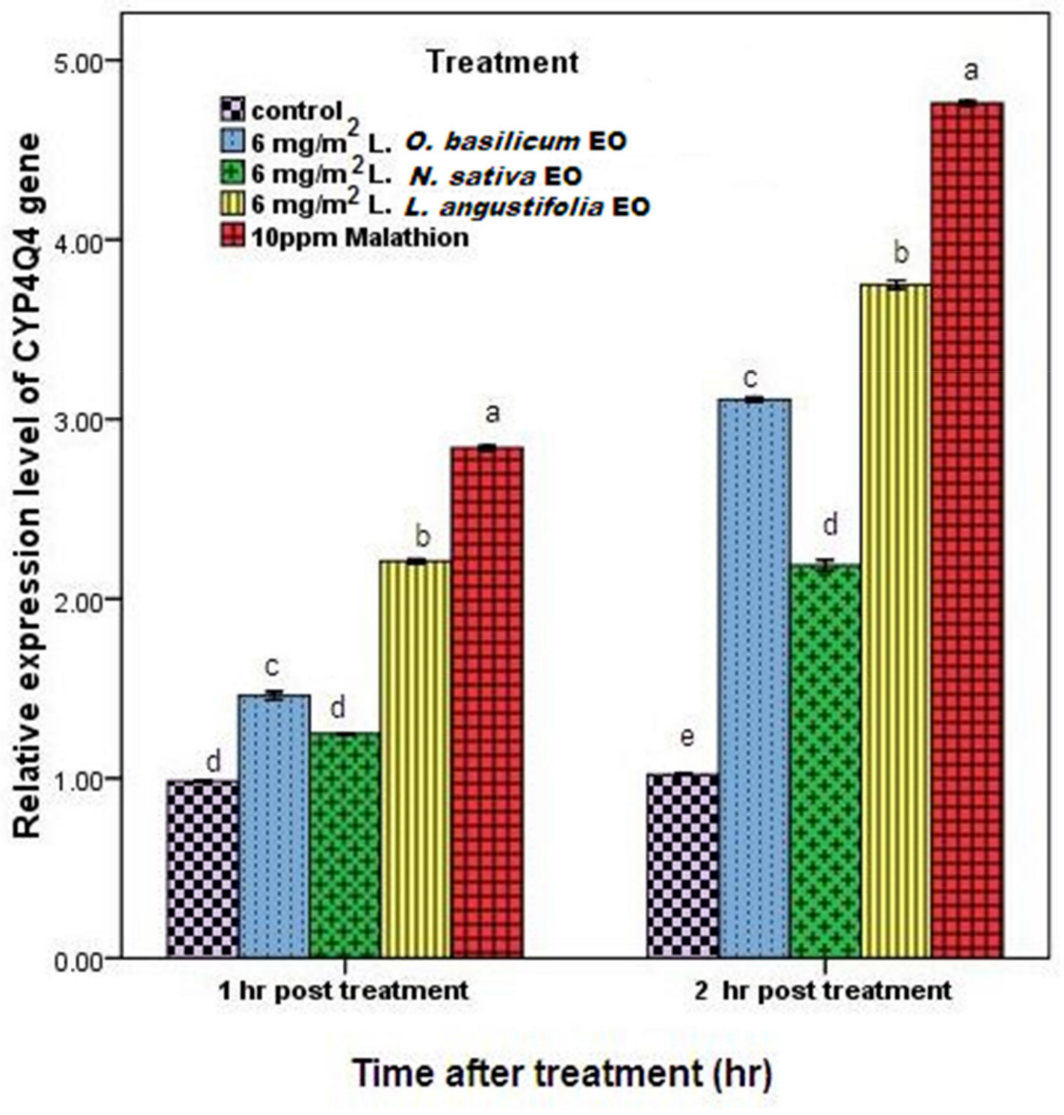
Relative gene expression of CYP4Q4 gene in *S. oryzae* adults after 1 and 2 h treated with 6 mg/cm^2^ of *O. basilicum, N. sativa* and *L. angustifolia* EOs (mean ± SD) compared with standard chemical insecticide (10 ppm malathion). Different letters indicate significant differences between different treatments at *p* < 0.05.

According to the qRT-PCR results, Figure 8 shows the maximum up-regulated expression level of CYP4Q4 cytochrome gene as 4.76 at 10 ppm malathion after 2 h changes in mRNA in *S. oryzae* adult treated with (6 mg/cm^2^) for 2 h, followed by 3.75 fold change in adult insect treated with (6 mg/cm^2^) of *L. angustifolia* EOs, and the minimum expression level reached 1.73 fold in adult insect treated with (6 mg/cm^2^) of *N. sativa* EOs for 1 h as compared with reference gene (housekeeping gene, β-Actin).

**Figure 9:**
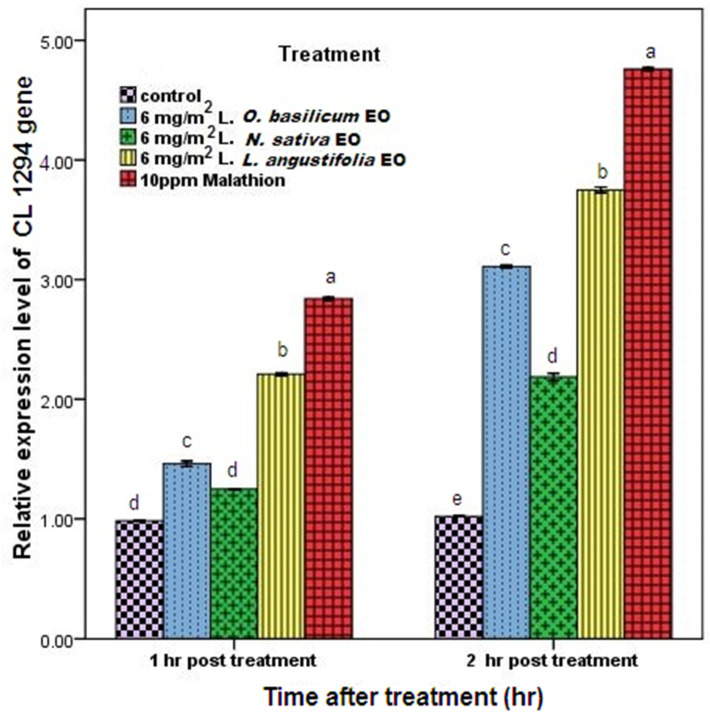
Relative gene expression of CL 1294 gene in *S. oryzae* adults after 1 and 2 h treated with 6 mg/cm^2^ of *O. basilicum, N. sativa,* and *L. angustifolia* EOs (mean ± SD) compared with standard chemical insecticide (10 ppm malathion). Different letters indicate significant differences between different treatments at *p* < 0.05.

According to the qRT-PCR results, Figure 9 reports that the maximum up-regulated expression level of CL 1294 gene exhibits 8.32 fold changes in mRNA in *S. oryzae* adult treated with (6 mg/cm^2^) of *L. angustifolia* EO for 2 h, followed by 4.76 at 10 ppm malathion after 2 h and the minimum expression level reached 2.08 fold in adult insect treated with (6 mg/cm^2^) concentration of *N. sativa* EO for 1 h as compared with reference gene (housekeeping gene, β-Actin).

## 4. Discussion

This study assesses the toxicity effect of three selected EOs (basil, black seeds, and Lavender) against *S. oryzae*. The highest insecticidal activity was recorded using basil and lavender EOs with 100% mortality effect. Pesticide disinfection is the most important method for the protection of the stored cereals and grains against insects [46]. Several research studies showed insecticides and repellence activities of many EOs extracted from different wild, spice, and herb plants against several stored-product insect pests [47,48,49,50,51,52]. In this study, Lavender EO had the highest toxicity activity for rice weevils with 100% mortality effect at 6 mg/cm^2^ at 12 and 48 h exposure time, in addition to the basil EO also had the highest mortality percentage 100% at 6 mg/cm^2^ at 12 h, this result was in agreed with [53], who reported the toxicity effect of basil, fennel, and geranium EOs against *S. oryzae* and *C. maculatus* through assessment of repellence and progeny production. Quick repellence activity in this study was highest for basil EO that recorded 61.2% for 0.75 mg/cm^2^ after 1 h against adult *S. oryzae* where repellence reached 82% after 5 h, theses result like results of [54], that reported repellence against *S. oryzae* after 1 h was 91.1%.

Active compounds in highest concentrations in basil EO were eugenol, linalool, estragole and methyl cinnamate, while active compounds in lavender were lavandulyl acetate, octacosane, and eugenol; black seeds characteristic by Limonene and 9, 12 Octadecadienoic acid active compounds. Eugenol active compound had repellent activity against *Ixodes ricinus* [55]. The estragole active compound had insecticide and pesticide activity against stored Vigna pest (*Callosobruchus maculatus*) [56]. Presences of estragole and t-anethole compounds in essential oil were an indicator for insecticide characters for this EO which had antimicrobial activity [57].

Eugenol is considered the important active compound in basil which had an effective effect against *S. zeamais* and *T. castaneum*, also had important fumigant activity with stored rice against the rice weevil [58]. The essential oils, especially basil and clove, can be used as an effective control agent for stored grain pests by fumigation. Active compounds in botanical EOs have some limitations such as low bioavailability, high volatility, and photodegradation that restrict their use on several occasions [59].

Previous reports are available on the fumigation activity related to various concentrations of plant EOs against pest insects *S*. *zeamais* [60] while the fumigant effect of investigated EOs in *S*. *zeamais* was enhanced by increasing the dose or exposure time of EOs, or different pest insects used [61,62]. The highest fumigant efficiency of clove and thyme EOs reported by [63], showed a 100% mortality of *S*. *oryzae*. Additionally, the results of [64] indicated that the EOs of clove caused 100% mortality similar to our result for basil and lavender Eos.

The repellant bioassay results in *S. oryzae* adults’ response to EOs using filter paper method show strong repellant effect (98.1%) after 48 h at a dose (0.75 mg/cm^2^*)* of *L. angustifolia* EOs, and 82.3% repellent after 48 h exposure to (0.75 mg/cm^2^) of *O. basilicum* EOs, as compared with 93.2% repellency after 48 h of exposure to (4 ppm) of chemical insecticide malathion. The essential oil of *O. basilicum* was principally composed of the monoterpenoid as terpinene and the terpene with alcohol group in *O. basilicum* and *N. sativa* Eos, such as linalool and linalyl acetate, which are known to have repellent and toxic activities against stored product insect [65]. The main components obtained in *O. basilicum* oil were similar to those described by [66,67,68]. Our results are similar to the previous study, the insecticidal toxicity in different pests by exposure to different concentrations of *C. cyminum* and *L. angustifolia* EOs [69,70,71,72,73]. According to phytochemical analysis data, the insecticidal activity related to the presence of linalyl acetate and linalool and other bioactive compounds in investigated *O. basilicum, N. sativa,* and *L. angustifolia EOs*, our phytochemical results were similar to previous studies, showing strong insecticides and repellent activities toxicity of *C. cyminum* and *L. angustifolia* EOs against different stored-product insects related to the presence of linalyl acetate and linalool [49,74,75]. Moreover, the fumigant toxicity of 1,8-cineole and linalool have been investigated against *B. germanica* (L.) and *O. surinamensis* by Abdelgaleil et al. [76]. In addition, [77] showed acetylcholine esterase inhibition in *S. oryzae* adults and *T. castaneum* larvae related to 1,8-cineole and linalool exposure.

The effective potentiality of studied plant EOs due to the presence of the most toxic components for insects such as linalool and linalyl acetate for *O. basilicum*, linalool, methyl ether, linoleic acid ethyl ester for *N. sativa* and lavandulyl acetate for *L. angustifolia* similar to previous studies proved major constituents in essential oils with toxic activity and insecticidal potentialities such as limonene, camphor, 1, 8-cineole, and g-terpinene [78,79]. Studied EOS resulted in death for *S. oryzae* adult pest, and this may be because of neurotoxic effect for these plants Eos, as revealed previously, which medicinal volatile and aromatic wild plants contain essential chemical constituents acting as inhibitors for *S. oryzae* and other insects [80,81]. In this study, toxicity against *S. oryzae* through detecting the mortality percentage was highest using *L. angustifolia* EO with the value of 100% at 48 and 24 h, this result agrees with the result of [82], who reported the highest mortality against *Sitophilus oryzae* and *Callosobruchus chinensis* with the value of 100% after two days using *Cinnamomum sieboldii* bark extract. The highest Inhibition activity and toxicity were recorded using EOs of *L. angustifolia* and *O. basilicum* due to their plants are aromatic and contain volatile oils. In the same context, [83] ensured that oils with high volatility led to the decay of insects through their fumigant and gaseous actions. Our results are in harmony with [84], who reported that cinnamon EO had the most toxic effect at 1 h against *S. zeamais*. Gene CYPs is a metabolic component in microorganisms and insects and plants; which released against any stresses as insecticides, pesticides, fungicides, and herbicides through increasing the activity of flavonoids compounds and increasing the antioxidant activity. Mode of action of CYPs occurs through increasing the content of CBT-ol which induces and exhibits resistance [85]. Results of qRT-PCR revealed the expression of selected genes CL8, CYP 4Q4, and CL1294 demonstrated the expression of these genes up and down in each treatment. Mode of action for detoxification genes inside insects has three pathways [86]. For pathway 1 functional group as nucleophilic was infused to the xenobiotic compound which active and considered as a water-soluble compound, the gene of cytochrome CYP 4Q4 is important in phase I. These genes have the main role in the detoxification and metabolization of insecticides leading to reduce toxic effect [87]. For phase II, enzymes increase solubility for water for the metabolite of Phase I through union with endogenous compound, which prevents tissue damage through combination with molecules of insecticides and CL1294 important in this phase [88]. Finally, in phase III, ABC enzyme transport xenobiotic compound out of the cell [89].

## 5. Conclusions

In conclusion, the highest repellence effects against *S. oryzae* were recorded by using basil EO with all concentrations and at different exposure times. This effect is related to the chemical composition of basil essential oil containing eugenol, linalool, and estragole. Lavender and basil EOs have the highest mortality effect against rice weevils. Expression of detoxification system of *S. oryzae* genes was increased in case of using lavender and basil EO. This study recommends using basil and lavender EOs as insecticides and pesticides against *S. oryzae* as biological control.

## Figures and Tables

**Table 1:** Nucleotide primer sequences used in qRT-PCR analysis in *S. oryzae* adult stage treated with essential oils.

Technique	Target Gene	Sequences (5′–3′)	Annealing °C
qRT-PCR	β-Actin-F	GACCTCTATGCCAACACAGT	60 °C
	β-Actin-R	AGTACTTGCGCTCAGGAGGA	
	CL8-F	CATCCGCAAACACAACAAAC	
	CL8 -R	TACCTGAAGGGTCCATATGG	
	CYP 4Q4-F	CAGTTTGGTGATTCAGATGATG	
	CYP 4Q4-R	GCACATCTGGGGACAAACTT	
	CL1294 –F	GTCTATGCACCTGGGTCGTT	
	CL1294 –R	GTCGGCAGACAAGGAAGACA	

**Table 2:** Phytochemical analysis of basil, black seeds and lavender EOs.

S. No.	Phytochemical Test	*O. basilicum*	*N. sativa*	*L. angustifolia*
1	Carbohydrate	+	-	-
2	Glycosides	+	+	-
3	Steroids	+	+	+
4	Terpenoids	+	+	+
5	Tannins	+	+	+
6	Sterols	+	+	+

**Table 3:** Chemical composition of *O. basilicum* EOs.

Quantitative ID	Component Identified	RI	LRI	Area (%)	Identification
1	Glycolaldehyde	873	923	3.27	RI, MS
2	Trioxane	686	660	t	RI, MS
3	Methyl formate	484	386	t	RI, MS
4	2-Chloroethanol	688	680	t	RI, MS
5	Acetic acid, 2-hydroxy-, ethyl ester	829	918	t	RI, MS
6	Acetic acid, hydrazide	863	946	1.14	RI, MS
7	(1S)-Camphor	1121	1120	30.321	RI, MS
8	Ethyl 3-(6-methoxy-3-methyl-2-benzofuranyl)-3-(p-methoxyphenyl) propionate	2749	2838	t	RI, MS
9	O-Ethyl S-[1-(4-methylphenyl)-2,3-diphenyl-2-cyclopropen-1-yl] carbonodithioate	3401	3490	t	RI, MS
10	1-{2,4-Bis[(trimethylsilyl)oxy]phenyl}-2-{4-[(trimethylsilyl)oxy]phenyl}-1-propanone	2631	2720	t	RI, MS
11	Octamethylcyclotetrasiloxane	827	994	9.936	RI, MS
12	2-Butyl-9(10H)-acridinone	2342	2431	t	RI, MS
13	Cridanimod	2354	2443	t	RI, MS
14	10-Butyl-10H-acridin-9-one	2169	2258	0.31	RI, MS
15	5-Amino-2-trimethylsilyloxy-acetophenone	1639	1728	1.311	RI, MS
16	1-Acetyl-1,5-diazacycloheptadecan-6-one	2550	2639	t	RI, MS
17	2,2-Bis[4-(dimethylamino)phenyl]-1-phenylethanone	2835	2924	36.630	RI, MS
18	Bis(3,4-dimethylphenyl) isophthalate	3040	3087	t	RI, MS
19	2,5-Diphenyl-1,2-dihydro-3H-1,2,4-triazole-3-thione	2483	2572	t	RI, MS
20	Triamterene	2829	2912	13.042	RI, MS
21	2-Amino-7-benzyl-4(1H)-pteridinone	2525	2614	t	RI, MS
22	1,2-Dihydrobenzo[b]fluoranthene	2246	2420	t	RI, MS
23	9-Phenanthrylmethyl 2,6-dimethylbenzoate	3062	3109	2.84	RI, MS
24	Propyl 2-tridecyn-1-yl terephthalate	2849	2896	nd	RI, MS
25	2,3-Dichlorophenyl 2-fluoro-6-(trifluoromethyl)benzoate	1916	2005	t	RI, MS
26	2,4-Dichloro-6-formylphenyl 2-fluoro-5-(trifluoromethyl)benzoate	2218	2307	nd	RI, MS
27	9-Phenanthrylmethyl 2,6-dimethylbenzoate	3062	3109	t	RI, MS
28	Nonyl N-(4-ethylbenzoyl)glycinate	2667	2756	t	RI, MS
29	Undecyl N-(4-ethylbenzoyl)glycinate	2866	2955	t	RI, MS
30	Tridecyl N-(4-ethylbenzoyl)glycinate	3065	3154	t	RI, MS
31	Isobutyl N-(4-ethylbenzoyl)glycinate	2106	2195	t	RI, MS
32	1,1′-(2,4,6-Trihydroxy-1,3-phenylene)di(1-propanone)	2238	2327	t	RI, MS
33	Propyl 2-fluoro-5-(trifluoromethyl)benzoate	1181	1270	t	RI, MS
34	1-{2,4-Bis[(trimethylsilyl)oxy]phenyl}-2-{4-[(trimethylsilyl)oxy]phenyl}-1-propanone	2631	2720	t	RI, MS
35	1-{2,4-Bis[(trimethylsilyl)oxy]phenyl}ethanone	1625	1714	t	RI, MS
36	Bis(2,5-dimethylphenyl) isophthalate	3040	3087	t	RI, MS
37	Pyridazinone	885	968	0.518	RI, MS
38	4-Pyridazinol	1122	1211	nd	RI, MS
39	Decahydroquinoline	1247	1330	nd	RI, MS
40	N,N,2-Trimethyl-3-butyn-2-amine	680	763	nd	RI, MS
41	Allylcyclohexylamine	1168	1251	nd	RI, MS
42	1,5-Bis(3-ethylphenoxy)-1,1,3,3,5,5-hexamethyltrisiloxane	2422	2511	nd	RI, MS
43	1,5-Bis(2,5-dimethylphenoxy)-1,1,3,3,5,5-hexamethyltrisiloxane	2450	2539	nd	RI, MS
44	1,1,3,3,5,5,7,7,9,9,11,11-Dodecamethylhexasiloxane	1341	1430	t	RI, MS
45	2,3,4,5-Tetraethyl-7,7-diphenylbicyclo[4.1.0]hepta-2,4-diene	2743	2798	t	RI, MS
46	1,1,3,3,5,5,7,7,9,9,11,11,13,13-Tetradecamethylheptasiloxane	1526	1615	t	RI, MS
47	Eugenol	1392	1364	17.52	RI, MS
48	Linalool	1082	1100	12.31	RI, MS
49	Estragole	1172	1178	7.20	RI, MS
50	Linalyl acetate	1272	1254	30.43	RI, MS

RI: Retention, LRI: Literature retention index, t: Trace (<0.05%), nd: not detected, MS: Mass spectrometry (GC/MS).

**Table 4:** Chemical composition of *N. sativa* EOs.

Quantitative ID	Component Identified	RI	LRI	Area (%)	Identification
1	β-Thujene	873	920	t	RI, MS
2	α-Thujene	902	926	2.061	RI, MS
3	α-Phellandrene	969	1005	t	RI, MS
4	p-Cymene	1042	1021	7.244	RI, MS
5	Thymoquinone	1340	1276	1.145	RI, MS
6	4,4a,5,6,7,8-Hexahydro-4a-methyl-2(3H)-naphthalinone	1357	1414	t	RI, MS
7	4-(3-Methyl-2-butenyl)-4-cyclopentene-1,3-dione	1397	1454	t	RI, MS
8	2(5H)-Furanone, 4-(2,3-dimethyl-2-buten-4-yl)-5-methoxy-	1493	1582	t	RI, MS
9	(11E,13Z)-1,11,13-Hexadecatriene	1618	1657	t	RI, MS
10	9-Hexadecenal	1808	1805	t	RI, MS
	Pentadecanoic acid	1869	1865	t	RI, MS
11	Palmitic acid	1968	1963	9.936	RI, MS
12	cis-10-Heptadecenoic acid	2075	2073	t	RI, MS
13	Methyl linoleate	2093	2087	t	RI, MS
14	Oleic acid chloride	2131	2220	0.31	RI, MS
15	Stearic acid	2167	2161	1.311	RI, MS
16	Oleic Acid	2175	2171	t	RI, MS
17	Linoleic acid	2183	2134	56.630	RI, MS
18	cis-11-Eicosenoic acid	2374	2362	t	RI, MS
19	2-Chloroethyl linoleate	2418	2458	t	RI, MS
20	Erucic acid	2572	2546	13.042	RI, MS
21	1-Oleoyl-rac-glycerol	2689	2714	t	RI, MS
22	2-Oleoylglycerol	2705	2780	t	RI, MS
23	Trielaidin	6149	6189	8.628	RI, MS

RI: Retention index, LRI: Literature retention index, t: Trace (<0.05%), MS: Mass spectrometry (GC/MS).

**Table 5:** Chemical composition of *L. angustifolia* EOs.

Quantitative ID	Component Identified	RI	LRI	Area (%)	Identification
1	Hexanal	806	819	10.44	RI, MS
2	Cyclobutanol	828	668	t	RI, MS
3	Eucalyptol	1059	1030	8.940	RI, MS
4	Trifluoroacetyl-.alpha.-terpineol	1167	1167	t	RI, MS
5	Lavandulyl acetate	1270	1273	19.24	RI, MS
6	Linalool acetate	1272	1261	t	RI, MS
7	Eugenol	1392	1356	29.35	RI, MS
8	Isoeugenol	1410	1451	t	RI, MS
9	Linalyl butyrate	1471	1422	t	RI, MS
10	Bicyclo[10.1.0]tridec-1-ene	1472	1472	14.69	RI, MS
	Hexyl cyclohexanecarboxylate	1544	1509	nd	RI, MS
11	Eugenol acetate	1552	1523	t	RI, MS
12	8-Hexadecyne	1629	1629	1.031	RI, MS
13	9,12-Tetradecadien-1-ol, (Z,E)-	1672	1677	t	RI, MS
14	9-Hexadecyn-1-ol	1872	1863	t	RI, MS
15	9-Hexadecenoic acid	1976	1953	0.65	RI, MS
16	Cyclohexanecarboxylic acid	2031	2084	nd	RI, MS
17	1,6-Octadien-3-ol, 3,7-dimethyl-, 2-aminobenzoate	2157	2175	15.35	RI, MS
18	Vaccenic acid	2175	2141	t	RI, MS
19	Cyclohexanecarboxylic acid, 2-tridecyl ester	2176	2168	nd	RI, MS
20	Linoleic acid	2183	2128	12.76	RI, MS
21	Oxacycloheptadec-8-en-2-one	2246	2206	t	RI, MS
22	cis-10-Nonadecenoic acid	2274	2225	t	RI, MS
23	Cyclohexanecarboxylic acid, 2-tetradecyl ester	2275	2267	nd	RI, MS
24	2-cis,cis-9,12-Octadecadienyloxyethanol	2344	2344	t	RI, MS
25	9-Decenyl laurate	2365	2365	nd	RI, MS
26	cis-11-Eicosenoic acid	2374	2362	13.48	RI, MS
27	Erucic acid	2572	2546	0.23	RI, MS
28	cis-13,16-Docasadienoic acid	2580	2566	t	RI, MS
29	Isocaryophyllene	1434	1427	2.67	RI, MS

RI: Retention index, LRI: Literature retention index, t: Trace (<0.05%), nd: not detected, MS: Mass spectrometry (GC/MS).

**Table 6:** Total phenol content (TPC), total flavonoid content (TFC) and free radical scavenging capacity (DPPH) from basil, black seeds and lavender EOs.

Phytochemistry	Basil	Black Seeds	Lavender
TPC (mg GAE/g)	31.4	17.8	23.4
TFC (mg RE/g)	17.6	9.7	12.5
DPPH (%)	18.9	16.4	11.2

## Data Availability

Relevant data applicable to this research are within the paper.
